# Paricalcitol Attenuates 4-Hydroxy-2-Hexenal-Induced Inflammation and Epithelial-Mesenchymal Transition in Human Renal Proximal Tubular Epithelial Cells

**DOI:** 10.1371/journal.pone.0063186

**Published:** 2013-05-17

**Authors:** Chang Seong Kim, Soo Yeon Joo, Ko Eun Lee, Joon Seok Choi, Eun Hui Bae, Seong Kwon Ma, Suhn Hee Kim, JongUn Lee, Soo Wan Kim

**Affiliations:** 1 Department of Physiology, Chonnam National University Medical School, Gwangju, Korea; 2 Department of Internal Medicine, Chonnam National University Medical School, Gwangju, Korea; 3 Department of Physiology, Chonbuk National University Medical School, Jeonju, Korea; National Cancer Institute, United States of America

## Abstract

4-Hydroxy-2-hexenal (HHE), the aldehyde product of lipid peroxidation, may be responsible for the pathogenesis of progressive renal disease. Recently, paricalcitol (19-nor-1,25-dihydroxyvitamin D2) was shown to be renoprotective through its anti-inflammatory and antifibrotic effects in various experimental nephropathy models. In this study, we investigated the effects of paricalcitol on inflammation and epithelial-mesenchymal transition (EMT) after HHE-induced renal tubular epithelial cell injury. To investigate the molecular mechanisms underlying HHE-induced renal tubular cell injury, the human proximal tubular epithelial (HK-2) cells cultured with 10 µM HHE in the presence or absence of paricalcitol. In HK-2 cells, paricalcitol attenuated the HHE-induced expression of extracellular signal-regulated kinase, c-Jun N-terminal kinase, and p38 mitogen-activated protein kinase, and prevented nuclear factor-κB (NF-κB) activation. The expression of the inflammatory proteins inducible nitric oxide synthase and cyclooxygenase-2 was attenuated by paricalcitol pretreatment. In addition, HHE increased the expression of the transforming growth factor (TGF)-β/Smad signaling proteins and fibrotic proteins, such as α-smooth muscle actin and connective tissue growth factor; this inducible expression was suppressed by pretreatment with paricalcitol. Treatment with HHE resulted in the activation of the β-catenin signaling pathway, and paricalcitol pretreatment reduced the expression of β-catenin in HHE-treated HK-2 cells. Coimmunoprecipitation shows that paricalcitol induced vitamin D receptor (VDR)/β-catenin complex formation in HK-2 cells. Also immunofluorescence staining revealed that co-localization of VDR and β-catenin in the nuclei. ICG-001, an inhibitor of β-catenin, decreased the expression of TGF-β1 and attenuated HHE-induced tubular EMT. These results show that paricalcitol attenuated HHE-induced renal tubular cell injury by suppressing inflammation and EMT process through inhibition of the NF-κB, TGF-β/Smad, and β-catenin signaling pathways.

## Introduction

Oxidative stress, which can often result in tissue damage, is defined as an imbalance between an excessive generation of oxidant compounds and insufficient antioxidant defense mechanisms. Oxidation of lipids by reactive oxygen species (ROS) generates several reactive molecules; of these, aldehydes are major end products that may result in severe oxidative injury since they have greater stability than ROS [1]. The kidney is particularly susceptible to lipid peroxidation since an abundance of long-chain polyunsaturated fatty acids in the composition of renal lipids make the kidney vulnerable to chain reactions mediated by free radicals [2]. In experimental models, lipid peroxidation of the proximal renal tubule has been suggested to play a role in renal carcinogenesis [3]. Furthermore, several studies have reported that reactive lipid-derived aldehydes induced Bax- and Bcl-2-dependent apoptosis [4], [5], [6]. Although malondialdehyde has been considered as the major deleterious lipid peroxidation byproduct, much of the current work on the toxicity of lipid peroxidation byproducts focuses on the more reactive hydroxyalkenals, such as 4-hydroxy-2-nonenal and 4-hydroxy-2-hexenal (HHE). These products are readily generated in vivo following application of various biological/biochemical insults [1]. Our more recent study demonstrated that HHE, an aldehyde product of lipid peroxidation, induces apoptosis through ROS-mediated activation of the nuclear factor-κB (NF-κB) pathway in human proximal epithelial (HK-2) cells [7]. Furthermore, increased ROS has been shown to be associated with kidney fibrosis, promoting the production of collagen, fibronectin, and α-smooth muscle actin (SMA) [8], and to play a pivotal role in inflammation through the NF-κB pathway [9]. However, it remains largely unknown whether lipid-derived aldehydes induce inflammatory responses and epithelial-mesenchymal transition (EMT) in tubular epithelial cells.

Several recent studies have indicated that paricalcitol (19-nor-1,25-hydroxy-vitamin D2), a synthetic vitamin D analog, has renoprotective effects, including anti-inflammatory and anti-fibrotic effects in various nephropathy models [10], [11], [12]. Moreover, Wnt/β-catenin signaling, which has been implicated in renal fibrosis, was inhibited by paricalcitol in a proteinuric animal model [13]. In the present study, we investigated the molecular mechanisms of inflammatory response and EMT and determined whether paricalcitol has anti-inflammatory and anti-fibrotic effects in HK-2 cells treated with HHE.

## Materials and Methods

### Cell Culture and Application of HHE and Paricalcitol to HK-2 Cells

Human renal proximal tubular epithelial cells, HK-2 (ATCC, Manassas, VA), were cultured. Briefly, cells were passaged every 3–4 days in 100-mm dishes containing combined Dulbecco’s modified Eagle’s medium (DMEM) and Hams F-12 medium (Sigma, St Louis, MO) supplemented with 10% fetal bovine serum (Life Technologies, Gaithersburg, MD, USA), 100 U/mL penicillin and 100 mg/mL streptomycin (Sigma). The cells were incubated in a humidified atmosphere of 5% CO2 and 95% air at 37°C for 24 h and sub-cultured at 70–80% confluence. For experimental use, HK-2 cells were plated onto 60-mm dishes in medium containing 10% fetal bovine serum for 24 h and cells were then switched to DMEM-F12 with 2% fetal bovine serum and incubated for an additional 16h. The cells were then treated with HHE (10 µM), either with or without paricalcitol (0.25 ng/ml; Abbott Laboratories, North Chicago, IL, USA). The control cells were treated with a buffer solution alone. The cells were harvested at the end of treatment for further analysis. HHE was obtained from Cayman Chemical, Inc. (Ann Arbor, MI).

### Nuclear Extracts Preparation

For nuclear extracts, cells were lysed using NE-PER® nuclear extraction reagent (NER) (Pierce Biotechnology, Rockford, IL) according to the manufacturer’s protocol. Briefly, HK-2 cells incubated with HHE were harvested by scraping into cold phosphate-buffered saline (PBS), pH 7.2 and then centrifuged at 14000 g for 2 min. After removing the supernatant, 100 µL of ice-cold cytoplasmic extraction reagent (CER) I was added to the dried cell pellets. After incubated on ice for 10 min, ice-cold CER II was added to the tube. The tube was centrifuged at 16000 g for 5 min and pellet fraction was suspended in 50 µL of ice cold NER. After centrifuging the tube at 16000 g for 10 min, the supernatant (nuclear extract) fraction was transferred to a clean tube [14], [15].

### Western Blot Analyses

The cells were harvested, washed twice with ice-cold PBS and re-suspended in lysis buffer (20 mM Tris–HCl, pH 7.4, 0.01 mM ethylenediaminetetraacetic acid, 150 mM NaCl, 1 mM phenylmethylsulfonyl fluoride, 1 µg/ml leupeptin, 1 mM Na_3_VO_4_) and sonicated briefly. After centrifugation, the supernatant was prepared as protein extract. The total protein concentrations were measured by bicinchoninic acid assay kit (Pierce; Rockford, IL, USA). Equal amounts of protein were separated on 9 or 12% sodium dodecyl sulfate polyacrylamide gels. The proteins were electrophoretically transferred onto nitrocellulose membranes (Amersham Pharmacia Biotech, Hybond ECL RPN3032D; Little Chalfont, UK) using Bio-Rad Mini Protean II apparatus (Bio-Rad, Hercules, CA). The blots were blocked with 5% milk in PBS-T (80 mM Na_2_HPO_4_, 20 mM NaH_2_PO_4_, 100 mM NaCl, and 0.1% Tween-20 at pH 7.5) for 1 h and then incubated overnight at 4°C with primary antibodies, followed by incubation with anti-rabbit or anti-mouse horseradish peroxidase-conjugated antibodies. The labeling was visualized by an enhanced chemiluminescence system.

### Primary Antibodies

The antibodies used were as follows: Anti-extracellular signal-regulated kinases 1/2 (ERK 1/2) (catalog number 9102), anti-phosphorylated ERK (p-ERK 1/2) (9101), anti-c-Jun N-terminal kinase (JNK) (9258), anti-phosphorylated JNK (p-JNK) (9251), anti-p38 mitogen-activated protein kinase (MAPK) (p38 MAPK) (8690) and anti-phosphospecific p38 MAPK (p-p38 MAPK) (4631), anti-glycogen synthase kinase (GSK)-3β (9135), transforming growth factor (TGF)-β1 (3711), phosphorylated Smad-2/3 (3101), Smad-4 (9515), Smad-6 (9519) and NF-κB p65 (3034; Cell Signaling Technology, Beverly, MA), PD98059 (a MEK inhibitor, 513000), SP600125 (a specific JNK inhibitor, 420119) and SB203580 (a p38 MAPK inhibitor, 559389) (Calbiochem, San Diego, CA), anti-inducible nitric oxide synthase (iNOS) (610328) and anti-β-catenin (610154; BD Transduction Laboratories, San Jose, CA), anti-cyclooxygenase-2 (COX-2) (160126; Cayman Chemical, MI), anti-vitamin D receptor (VDR) (SC-1008), connective tissue growth factor (CTGF) (SC-14939) and anti-IκBα (SC-371; Santa Cruz Biotechnology, Santa Cruz, CA), Histone H3 (9715; Cell Signaling Technology), α-SMA (A2547) and β-actin (A3854; Sigma) antibodies were commercially obtained.

### Coimmunoprecipitation

Coimmunoprecipitation was carried out by using an established method [16]. Briefly, HK-2 cells after various treatments were lysed on ice in 1 ml of nondenaturing lysis buffer that contained 1% Triton X-100, 0.01 M Tris-HCl (pH 8.0), 0.14 M NaCl, 0.025% NaN_3_, 1% protease inhibitors cocktail, and 1% phosphatase inhibitors cocktail I and II (Sigma). After preclearing with normal IgG, cell lysates (0.4 mg of protein) were incubated overnight at 4°C with 4 µg of anti-VDR, followed by precipitation with 40 µl of protein A/G Plus-agarose for 3 h at 4°C. The precipitated complexes were separated on sodium dodecyl sulfate polyacrylamide gels and immunoblotted with anti-β-catenin antibody.

### Immunocytochemical Staining

HK-2 cells were cultured on chamber slide (Nalge Nunc International) and treated with HHE (10 µM) with paricalcitol (0.25 ng/ml). Cells were stained with anti-VDR and double-stained with anti-β catenin. Alexa Flour 488-labeled (green) goat anti-mouse IgG and Alexa Flour 568-labeled (red) goat anti-rabbit IgG (Molecular Probes Invitrogen) were used as the secondary antibodies. Cells were fixed in 4% paraformaldehyde at room temperature for 20 min, permeabilized with 0.5% Triton X-100 for 20 min, washed with PBS, and blocked for 30 min at room temperature with blocking solution (5% bovine serum albumin). The primary antibodies rabbit anti-VDR and mouse anti-β-catenin were incubated with the cells overnight at 4°C in a humidified chamber. After washes, secondary antibody conjugated with either Alexa Flour 488 or Alexa Flour 568 (1∶200 dilution; Invitrogen) was incubated with chamber slide for 1 h at room temperature. To identify nuclei, cells were counterstained with 4,6-diamidino-2-phenylindole (Invitrogen) for 10 min. Stained cells were visualized using an AX70 fluorescence microscope (Olympus, Tokyo, Japan).

### Statistical Analysis

Results are expressed as mean ± standard error of the mean (SEM). Differences between groups were analyzed by one-way analysis of variance with *post-hoc* comparison. Differences with values of *p*<0.05 were considered significant.

## Results

### Cell Inflammation

**Figure 1:**
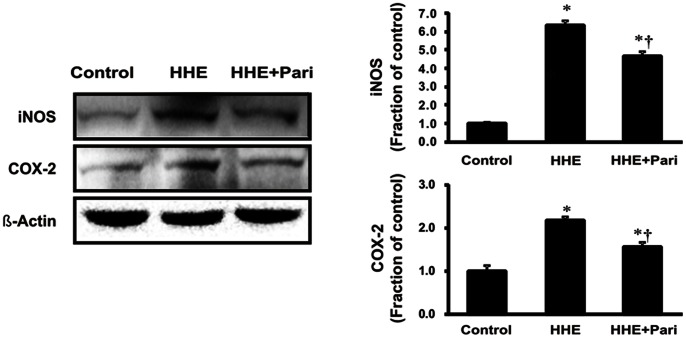
Effects of paricalcitol on iNOS and COX-2 expression in HK-2 cells incubated with HHE. Semiquantitative immunoblotting of iNOS and COX-2 is shown. iNOS and COX-2 expression were significantly increased by 10 µM HHE, and these changes were attenuated by paricalcitol pretreatment (0.25 ng/mL). *P<0.05 compared to the control; † *P*<0.05 compared to HHE-treated HK-2 cells. Pari, paricalcitol.

To determine the effects of HHE and paricalcitol on renal cell inflammatory response in HK-2 cells, we analyzed the protein expression of iNOS and COX-2 by immunoblotting in HK-2 cells following pretreated with paricalcitol (0.25 ng/mL) for 3 h and subsequent treatment with 10 µM HHE for 8 h. Both iNOS and COX-2 exhibited significantly increased expression after HHE treatment, and paricalcitol pretreatment prevented this HHE-mediated increase in iNOS and COX-2 expression (Figure 1).

### MAPK Expression

**Figure 2:**
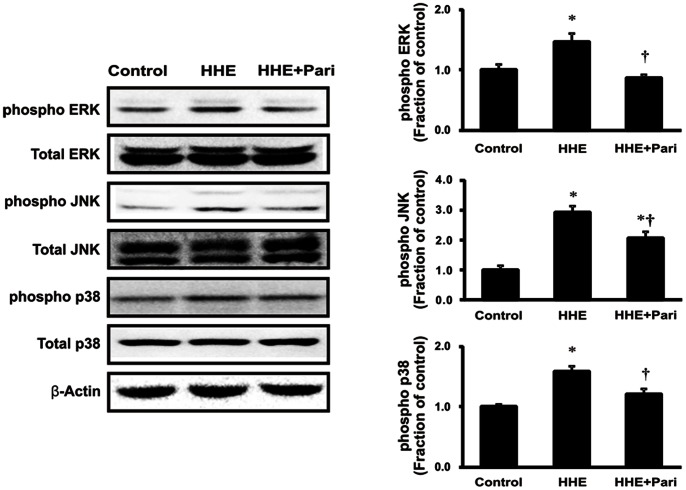
Effects of paricalcitol on the phosphorylation of ERK1/2, JNK, and p38 MAPK in HK-2 cells incubated with HHE. HK-2 cells were pretreated with paricalcitol (0.25 ng/mL) for 3 h prior to incubation with 10 µM HHE for 30 min. HHE treatment increased the levels of p-ERK1/2, p-JNK, and p-p38 MAPK, and paricalcitol pretreatment suppressed these effects. *P<0.05 compared to the control; † *P*<0.05 compared to HHE-treated HK-2 cells. Pari, paricalcitol.

**Figure 3:**
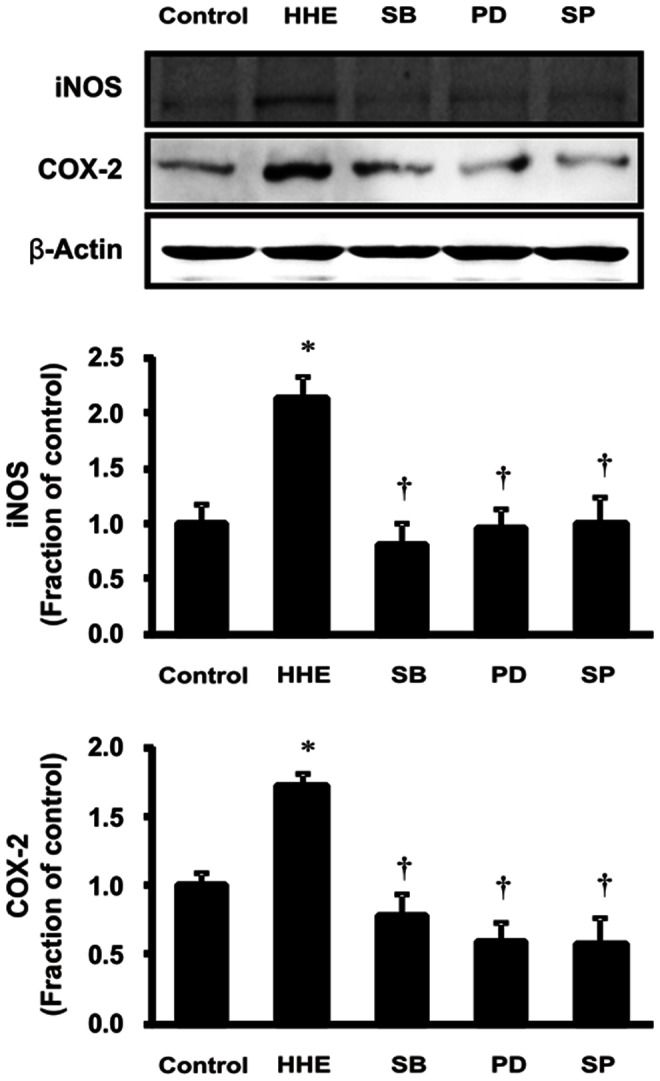
Suppression of the expression of iNOS and COX-2 by SB203580 (p38 MAPK inhibitor), PD98059 (ERK inhibition) and SP600125 (JNK inhibition). HK-2 cells were incubated with HHE (10 µM) for 8 hr after pretreatment for 1 h with SB203580, PD98059, SP 600125. *P<0.05 compared to the control; † *P*<0.05 compared to HHE-treated HK-2 cells.

As shown in Figure 2, phosphorylation of ERK1/2, JNK, and p38 MAPK increased following treatment with 10 µM HHE for 30 min in HK-2 cells. Similar to iNOS and COX-2, the observed HHE-dependent increased in p-ERK1/2, p-JNK, and p-p38 MAPK was suppressed by pretreatment with 0.25 ng/mL paricalcitol for 3 h. However, HHE did not affect the expression of total ERK1/2, JNK, or p38 MAPK. We determined the effects of specific inhibitors of ERK1/2 (PD98059), JNK (SP600125) and p38 MAPK (SB203580) on inflammation. All of these specific inhibitors attenuated the expression of iNOS and COX-2 after treatment with HHE (Figure 3). These findings suggest that MAPK pathway is involved in the HHE-induced inflammation.

### NF-κB Expression

**Figure 4:**
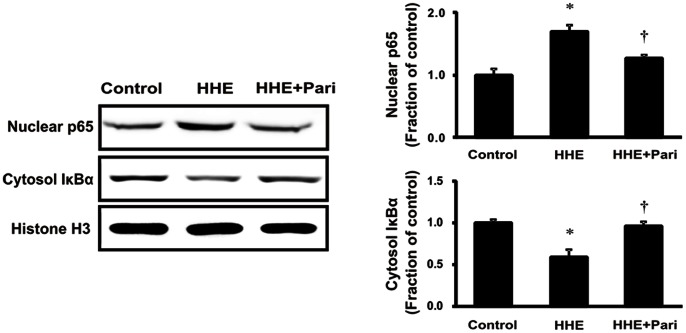
Effects of paricalcitol on the nuclear localization of the NF-κB p65 subunit and cytosolic total IκBα expression in HHE-treated HK-2 cells. Pretreatment with paricalcitol (0.25 ng/mL) for 3 h before exposure to 10 µM HHE for 1 h suppressed the overexpression of nuclear NF-κB p65 in HK-2 cells, while cytosolic total IκBα expression was restored the suppression in HHE-treated HK-2 cells after paricalcitol pretreatment. *P<0.05 compared to the control; † *P*<0.05 compared to HHE-treated HK-2 cells. Pari, paricalcitol.

Next, we investigated the effects of HHE and pretreatment with paricalcitol on the expression of the NF-κB p65 subunit in nuclear extracts and cytosolic total IκBα expression from HK-2 cells. After 1-h treatment with 10 µM HHE, HK-2 cells exhibited increased expression of the NF-κB p65 subunit, as compared with controls. NF-κB p65 subunit expression was also examined in HK-2 cells pretreated with 0.25 ng/mL paricalcitol for 3 hr followed by exposure to 10 µM HHE for 1 h. As expected, paricalcitol pretreatment reversed the HHE-dependent increase in NF-κB p65 subunit expression, while cytosolic total IκBα expression restored the suppression in HHE-treated HK-2 cells after paricalcitol pretreatment. These results are indicating that paricalcitol could mediate the inflammatiory response through regulation of NK- κB expression (Figure 4).

### EMT and TGF-β1/Smad Signaling

**Figure 5:**
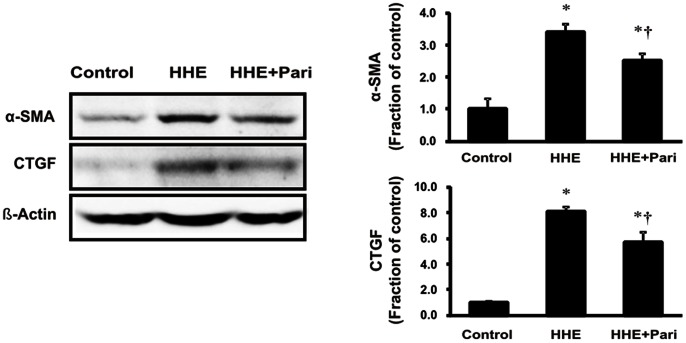
Effects of paricalcitol on the expression of α-smooth muscle actin (α-SMA) and connective tissue growth factor (CTGF). Treatment with 10 µM HHE for 6 h increased the expression of α-SMA and CTGF compared to control-treated HK cells, and this effect was prevented by pretreatment with paricalcitol for 3 h. *P<0.05 compared to the control; † *P*<0.05 compared to HHE-treated HK-2 cells. Pari, paricalcitol.

**Figure 6:**
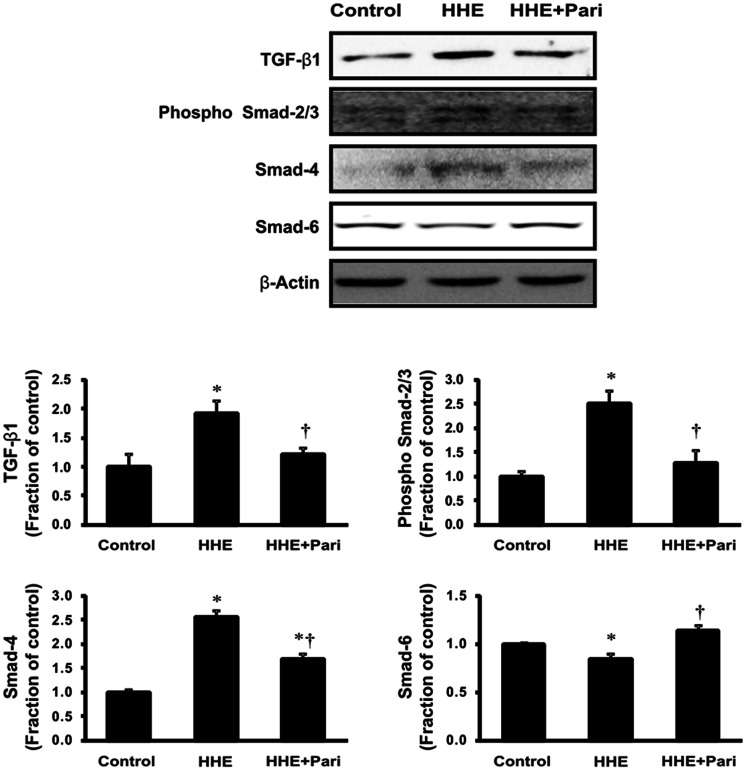
Effects of paricalcitol on TGF-β1 expression and Smad signaling in HHE-induced HK-2 cells. Semiquantitative immunoblotting for TGF-β1, phosphorylated Smad-2/3, Smad-4, and Smad-6 is shown. TGF-β1 expression increased significantly in HK-2 cells treated with 10 µM HHE for 6 h, and increases in phosphorylated Smad-2/3 and Smad-4 expression were accompanied by a decrease in inhibitory Smad-6, consistent with the changes in TGF-β1, indicating that TGF-β1 triggered the Smad signaling cascade. Pretreatment with paricalcitol (0.25 ng/mL) attenuated all of these changes. *P<0.05 compared to the control; † *P*<0.05 compared to HHE-treated HK-2 cells. Pari, paricalcitol.

As shown in Figure 5, we next examined the expression of α-SMA and CTGF to determine whether EMT of proximal tubular epithelial cell was induced following 6-h incubation with HHE. In HK-2 cells, 10 µM HHE enhanced the expression of α-SMA and CTGF, as compared with controls, and this effect was prevented by paricalcitol pretreatment. The expression of TGF-β1 also increased significantly in HHE-treated HK-2 cells, and this effect was similarly reversed by pretreatment with 0.25 ng/mL paricalcitol. We also examined the TGF-β1-mediated Smad signaling pathway by evaluating phosphorylated Smad-2/3 and Smad-4 levels as well as the expression of inhibitory Smad-6. Increases in phosphorylated Smad-2/3 and Smad-4 levels were accompanied by a decrease in inhibitory Smad-6 in HK-2 cells treated with 10 µM HHE for 6 h. On the contrary, pretreatment with 0.25 ng/mL paricalcitol prevented these changes (Figure 6).

### EMT and β-catenin Signaling

**Figure 7:**
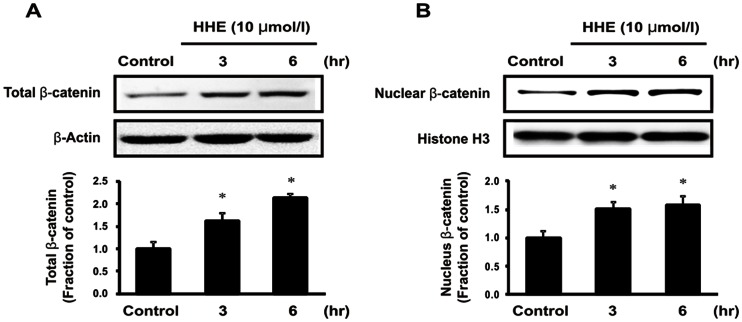
Expression of β-catenin. (A) Semiquantitative immunoblotting at 3 or 6 h after treatment with 10 µM HHE. The expression of total cellular β-catenin protein was increased at 3 h and continued to rise at 6 h in HHE-treated HK-2 cells. (B) Similarly, nuclear β-catenin was increased at 3 h and maintained at 6 h after HHE treatment. *P<0.05 compared to the control.

**Figure 8:**
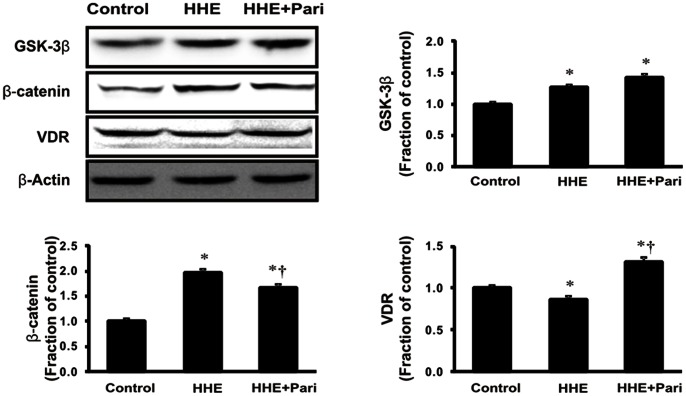
Effects of paricalcitol on the expression of β-catenin and the vitamin D receptor (VDR). The expression of β-catenin increased in HK-2 cells treated with 10 µM HHE for 6 h. In contrast, the expression of VDR decreased after HHE treatment. Paricalcitol pretreatment significantly attenuated these changes. The expression of glycogen synthase kinase-3β was not affected by paricalcitol pretreatment. *P<0.05 compared to the control; † *P*<0.05 compared to HHE-treated HK-2 cells. Pari, paricalcitol.

**Figure 9:**
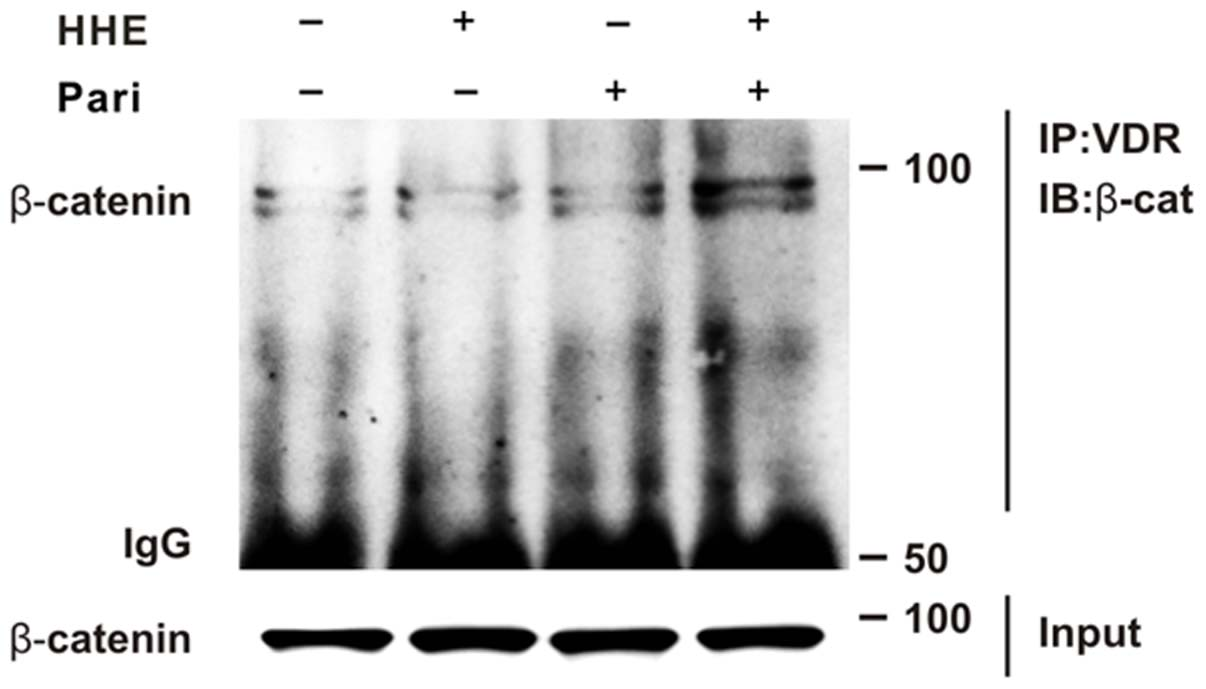
The sequestration of the β-catenin transcriptional activity, via vitamin D receptor (VDR), after paricalcitol treatment. Coimmunoprecipitation reveals that paricalcitol induced VDR/β-catenin complex formation in HK-2 cells. The cells were pretreated with paricalcitol for 3 h, followed by 10 µM HHE treatment for 6 h. Pari, paricalcitol.

**Figure 10:**
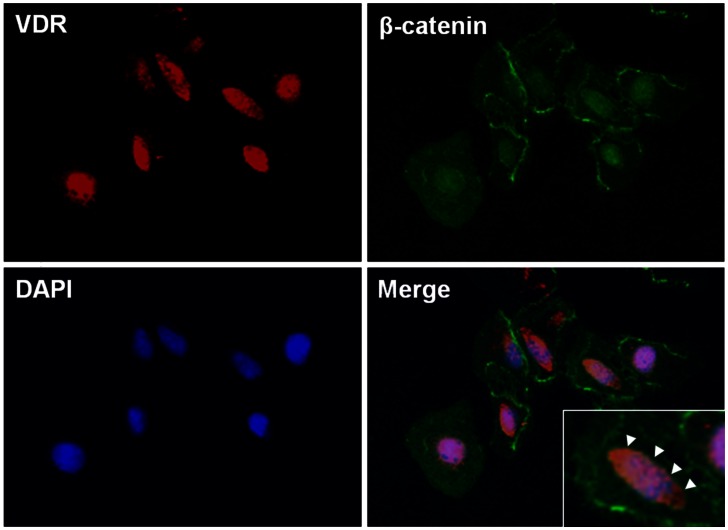
Nuclear co-localization of vitamin D receptor (VDR) and β-catenin in HK-2 cells. Immunofluorescence staining demonstrated that the expression of VDR (red) was found to be co-localized with β-catenin (green) in the nuclei. Nucleus was stained with 4,6-diamidino-2-phenylindole (blue). The cells were pretreated with paricalcitol for 3 h, followed by 10 µM HHE treatment for 6 h (original magnification x 200). Inset show nuclear co-localization of VDR and β-catenin at a higher magnification indicated by arrow heads.

Next, we assessed changes in β-catenin levels in both total cellular and nuclear extracts from HK-2 cells after incubation with 10 µM HHE for 3 or 6 h. Total cellular β-catenin expression was increased at 3 h and continued to rise at 6 h in HHE-treated HK-2 cells; similarly, the nuclear translocation of β-catenin was increased at 3 h and maintained at 6 h after HHE treatment (Figure 7). The HHE-induced increased in β-catenin expression in HK-2 cells was attenuated by pretreatment with 0.25 ng/mL paricalcitol. Moreover, while the expression of GSK-3β was not affected by paricalcitol pretreatment, HHE caused a reduction in VDR expression, and this reduction was unregulated by paricalcitol pretreatment (Figure 8). We next tested whether activated VDR interacts with β-catenin by using a coimmunoprecipitation method. Incubation of HK-2 cells with paricalcitol induced VDR to interact with β-catenin, as shown by increased VDR/β-catenin complex formation after HHE treatment (Figure 9). Also immunofluorescence staining revealed that nucleus co-localization of VDR and β-catenin in HK-2 cells. VDR was localized exclusively in the nuclei, whereas β-catenin was found to be present throughout the cells that were pretreated with paricalcitol for 3 h, followed by 10 µM HHE treatment for 6 h. Their co-expression mostly localized in the nuclei (Figure 10).

**Figure 11:**
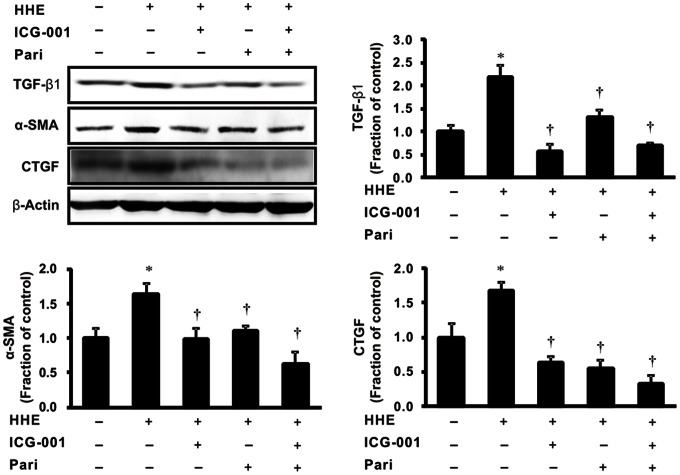
Effects of a β-catenin inhibitor (ICG-001) on tubular epithelial-mesenchymal transition. Semiquantitative immunoblotting revealed that pretreatment of 10 µM ICG-001 for 3 h significantly reduced the expression of TGF-β1, α-SMA, and CTGF after treatment with 10 µM HHE for 6 h. Similarly, paricalcitol (0.25 ng/mL) pretreatment attenuated these effects through the inhibition of HHE-induced TGF-β signaling. *P<0.05 compared to the control; † *P*<0.05 compared to HHE-treated HK-2 cells. Pari, paricalcitol.

To determine the association between β-catenin signaling and HHE-induced EMT, we used ICG-001, an inhibitor of β-catenin. Semiquantitative immunoblotting revealed that 10 µM ICG-001 attenuated the observed HHE-induced increases in TGF-β1, α-SMA, and CTGF expression in HK-2 cells, suggesting that β-catenin signaling is involved in HHE-induced renal tubular fibrosis (Figure 11).

### Effects of Paricalcitol Alone in HK-2 Cells

We have previously verified effects of paricalcitol treatment alone [17]. The expression of iNOS, COX-2, MAPKs, phosphoylated NF-κB p65 subunit, total IκB-α, TGF-β1, VDR, β-catenin, and EMT markers was not altered with paricalcitol alone compared to those of control cells (Figure S1).

## Discussion

In this study, we showed that HHE-induced oxidative stress enhanced the phosphorylation of ERK1/2, JNK, and p38 MAPK signaling pathways, resulting in nuclear translocation of NF-κB in renal tubular epithelial cells. Our findings are consistent with a previous study demonstrating that HHE activated ERK and p38 MAPK, leading to oxidative stress-associated activation of NF-κB in endothelial cells [18]. In our study, we further investigated HHE- and paricalcitol-mediated changes in other inflammatory components and signaling proteins. The NF-κB pathway does regulate pro-inflammatory cytokine production, leukocyte recruitment, and cell survival, all of which are important contributors to the inflammatory response [19]. Moreover, we also found that HHE increased the nuclear translocation of the NF-κB p65 subunit and promoted the upregulation of inflammation markers, such as iNOS and COX-2, in HK-2 cells. In contrast, paricalcitol attenuated the observed HHE-induced increases in renal iNOS, COX-2, and p65 NF-κB, suggesting that paricalcitol exerted an NF-κB-dependent anti-inflammatory effect on HHE-treated HK-2 cells via inactivation of the MAPK pathway.

Renal fibrosis is the principal process underlying the progression of chronic kidney disease to end-stage renal disease. Additionally, tubulointerstitial fibrosis has evolved as the most consistent predictor of the irreversible loss of renal function [20]. The EMT is defined as the sequential loss of epithelial markers and the new acquisition of mesenchymal markers. Recently, its role in renal interstitial fibrosis *in vivo* has been challenged by new cell fate tracing studies [21], [22], [23], which conflicts with earlier studies [24], [25]. Although there are no solid data supporting EMT as *in vivo* process in renal fibrosis, the EMT is still widely accepted as a mechanism by that contributes to the progression of renal fibrosis. TGF-β is a central mediator in the pathogenesis of renal fibrosis, mostly by inducing extracellular matrix (ECM) production and proliferation of myofibroblasts and fibroblasts, but also through immunoregulatory functions [26]. Also, the reduction of fibrosis in response to suppression of the TGF-β pathway reflected a blocking effect on EMT [27]. In this study, we showed that the expression of TGF-β1 was increased by HHE treatment, an affect that was ameliorated by paricalcitol pretreatment in HK-2 cells. The binding of TGF-β1 to its type II receptor can activate the TGF-β receptor type I-kinase, resulting in phosphorylation of Smad-2 and Smad-3. Consequently, phosphorylated Smad-2/3 binds to the common Smad-4 and forms the Smad complex, which translocates into the nucleus to regulate the transcription of target genes, including α-SMA and CTGF. Smad-6 and Smad-7 are inhibitory Smads that negatively regulate Smad-2/3 activation and function by targeting the type I TGF-β1 receptor and Smad for degradation via the ubiquitin proteasome degradation mechanism [28], [29]. Therefore, Smad signaling is a critical downstream pathway responsible for mediating the biologic effects of TGF-β1. In the present study, HK-2 cells treated with HHE exhibited an increase in the expression of phophorlyated Smad-2/3 and Smad-4, accompanied by a corresponding decrease in Smad-6 expression, suggesting that HHE induced renal tubular EMT. Importantly, these HHE-induced changes were effectively reversed by paricalcitol. These findings indicated that paricalcitol may have therapeutic effects, not only for renal tubular inflammation, but also for EMT in HHE-induced renal injury.

In addition, several studies have shown that the ERK and p38 MAPK pathways activate Smad signaling through TGF-β-independent or -dependent mechanisms [30], [31], [32]. Similarly, angiotensin II induces the Smad signaling pathway to stimulate ECM production through ERK/p38 MAPK and Smad cross-talk pathways in renal tubular cells [33]. Indeed, the signals controlling renal inflammation and fibrosis may engage in cross-talk. Therefore, in the present study, ERK1/2 and p38 MAPK signaling pathways may also appear to mediate HHE-induced renal tubular EMT.

The EMT is induced via major signaling events, including induction of TGF-β/Smad, integrin-linked kinase, and Wnt/β-catenin signaling pathways [34]. In this study, we determined the relationships between Wnt/β-catenin signaling and renal tubular EMT after HHE treatment; our data demonstrated that the expression of β-catenin was increased by treatment with HHE, and these changes were attenuated by paricalcitol pretreatment. However, HHE-treatment increased the expression of GSK-3β in HK-2 cells, which was not affected by paricalcitol pretreatment. It was supposed that paricalcitol treatment might directly affect the β-catenin level, not via GSK-3β level. A recent study showed that Wnt/β-catenin signaling, which can be inhibited by paricalcitol treatment, plays a critical role in promoting proteinuria and renal fibrosis [13]. In this pathway, paricalcitol may induce VDR binding to nuclear β-catenin, promoting the sequestration of β-catenin and suppressing its transcriptional activity in the nuclei. Another study indicated that vitamin D analogs appear to be mediated by ligand-activated VDR competing with transcription T cell factor-4 for β-catenin biding, enhancing the differentiation of colon carcinoma cells by inhibiting β-catenin signaling [35]. Indeed, paricalcitol reduced the overexpression of β-catenin and restored VDR expression in HK-2 cells after HHE treatment. It appears that paricalcitol, via VDR, effects on β-catenin signaling by sequestering β-catenin transcriptional activity. Our immunofluorescence experiments also showed that the expression of VDR and β-catenin were co-localized in the nuclei. In addition, the β-catenin inhibitor ICG-001 attenuated HHE-induced changes in the expression of α-SMA and CTGF and inhibited the TGF-β signaling pathway. Taken together, these data indicate that the β-catenin signaling pathway may mediate HHE-induced renal tubular EMT, which can be effectively inhibited by paricalcitol.

### Conclusions

The results presented in this study demonstrated that paricalcitol, a synthetic, low-calcemic vitamin D analog, exerted an impressive renal anti-inflammatory effect in HHE-treated HK-2 cells through inhibition of the NF-κB signaling pathway via inactivation of the MAPK pathway. Moreover, this compound also attenuated renal tubular EMT, a process in which TGF-β1 and β-catenin signaling play critical roles. Taken together, our data indicate that blocking these signaling pathways by paricalcitol treatment is a plausible strategy for therapeutic intervention in oxidative stress-induced renal injury.

## Supporting Information

Figure S1 **Semiquantitative immunoblotting in HK-2 cells treated with paricalcitol alone compared to controls.** The expression of iNOS, COX-2, β-catenin, VDR and α-SMA was not changed by paricalcitol.
(TIF)
