# Correction: Paricalcitol Attenuates 4-Hydroxy-2-Hexenal-Induced Inflammation and Epithelial-Mesenchymal Transition in Human Renal Proximal Tubular Epithelial Cells

**DOI:** 10.1371/annotation/ae0a1951-1b75-4a4f-865b-86acc4351139

**Published:** 2013-10-15

**Authors:** Chang Seong Kim, Soo Yeon Joo, Ko Eun Lee, Joon Seok Choi, Eun Hui Bae, Seong Kwon Ma, Suhn Hee Kim, JongUn Lee, Soo Wan Kim

The affiliation of the first, third, fourth, fifth, sixth and ninth authors is incorrect. Chang Seong Kim, Ko Eun Lee, Joon Seok Choi, Eun Hui Bae, Seong Kwon Ma, and Soo Wan Kim are not affiliated with the institution currently indicated in the first affiliation, but rather with the institution currently indicated in the second affiliation, Department of Internal Medicine, Chonnam National University Medical School, Gwangju, Korea.

In addition, the affiliation of the second and eighth authors is incorrect. Soo Yeon Joo and JongUn Lee are not affiliated with the institution currently indicated in the second affiliation, but rather with the institution currently indicated in the first affiliation, Department of Physiology, Chonnam National University Medical School, Gwangju, Korea.

For article indexing purposes, the following institution should be considered the first affiliation: Department of Internal Medicine, Chonnam National University Medical School, Gwangju, Korea. 

