# Supplementary figures and images for: Paricalcitol Attenuates 4-Hydroxy-2-Hexenal-Induced Inflammation and Epithelial-Mesenchymal Transition in Human Renal Proximal Tubular Epithelial Cells
Source: PLoS One. 2013 May 17;8(5):e63186. doi: 10.1371/journal.pone.0063186 (PMC3656901; doi:10.1371/journal.pone.0063186)

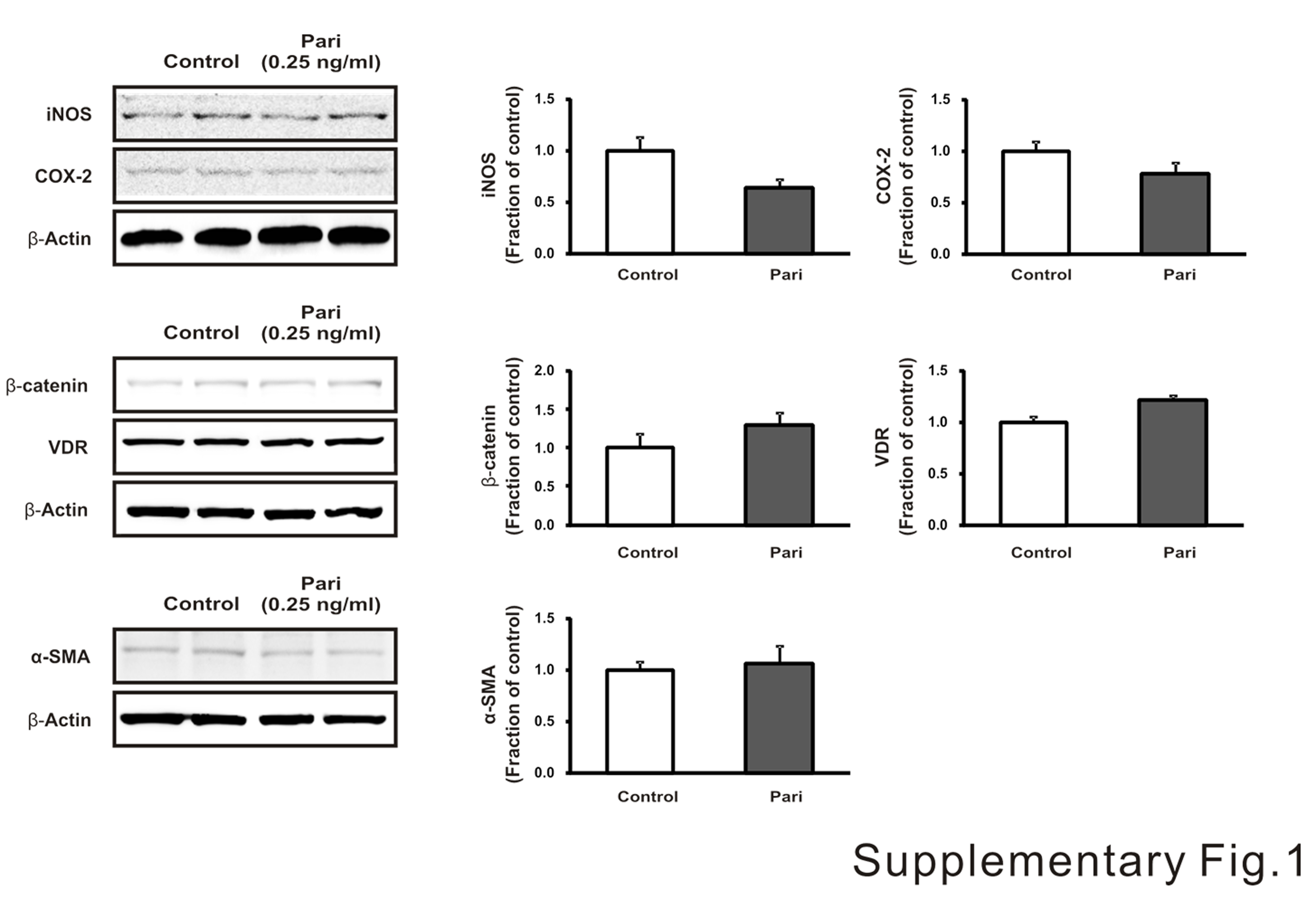

Supplement: Figure S1 — Semiquantitative immunoblotting in HK-2 cells treated with paricalcitol alone compared to controls. The expression of iNOS, COX-2, β-catenin, VDR and α-SMA was not changed by paricalcitol. (TIF) [file pone.0063186.s001.tif]
